# Chitosan in Mucoadhesive Drug Delivery: Focus on Local Vaginal Therapy

**DOI:** 10.3390/md13010222

**Published:** 2015-01-07

**Authors:** Toril Andersen, Stefan Bleher, Gøril Eide Flaten, Ingunn Tho, Sofia Mattsson, Nataša Škalko-Basnet

**Affiliations:** 1 Drug Transport and Delivery Research Group, Department of Pharmacy, Faculty of Health Sciences, University of Tromsø The Arctic University of Norway, Tromsø 9037, Norway; E-Mails: toril.andersen@uit.no (T.A.); bleher.stefan@gmail.com (S.B.); goril.flaten@uit.no (G.E.F.); 2 PharmaLuxLab Research Group, School of Pharmacy, Faculty of Mathematics and Natural Sciences, University of Oslo, 0316 Oslo, Norway; E-Mail: ingunn.tho@farmasi.uio.no; 3 Department of Pharmacology and Clinical Neuroscience, Division of Clinical Pharmacology, Umeå University, Umeå SE-90187, Sweden; E-Mail: sofia.mattsson@pharm.umu.se

**Keywords:** chitosan, drug delivery, mucoadhesion, vaginal therapy, FITC-dextran

## Abstract

Mucoadhesive drug therapy destined for localized drug treatment is gaining increasing importance in today’s drug development. Chitosan, due to its known biodegradability, bioadhesiveness and excellent safety profile offers means to improve mucosal drug therapy. We have used chitosan as mucoadhesive polymer to develop liposomes able to ensure prolonged residence time at vaginal site. Two types of mucoadhesive liposomes, namely the chitosan-coated liposomes and chitosan-containing liposomes, where chitosan is both embedded and surface-available, were made of soy phosphatidylcholine with entrapped fluorescence markers of two molecular weights, FITC-dextran 4000 and 20,000, respectively. Both liposomal types were characterized for their size distribution, zeta potential, entrapment efficiency and the* in vitro* release profile, and compared to plain liposomes. The proof of chitosan being both surface-available as well as embedded into the liposomes in the chitosan-containing liposomes was found. The capability of the surface-available chitosan to interact with the model porcine mucin was confirmed for both chitosan-containing and chitosan-coated liposomes implying potential mucoadhesive behavior. Chitosan-containing liposomes were shown to be superior in respect to the simplicity of preparation, FITC-dextran load, mucoadhesiveness and* in vitro* release and are expected to ensure prolonged residence time on the vaginal mucosa providing localized sustained release of entrapped model substances.

## 1. Introduction

Chitosan is a linear polysaccharide that is composed of copolymers of β(1-4)-linked *N*-acetylglucosamide and glucosamine. It is obtained by deacetylation of chitin, a natural polymer obtained from various sources, such as crustacean shells, fungi and bacteria; as a pharmaceutical raw material it is mostly obtained as a waste product of the shell fish industry, and is interesting as an affordable, renewable and sustainable product [1,2,3,4]. Chitosan can be obtained exhibiting various degrees of deacetylation (DD) and molecular weights, which determine its physicochemical and biological properties. The DD, as well as molecular weight, are directly proportional to physical properties, such as the solubility and viscosity. The mucoadhesiveness, antimicrobial effects and other biological properties are also related to the DD [5]. Although chitosan exhibits toxic effects on several bacteria, fungi and parasites, it is regarded safe for use in humans [6]. Chitosan is biodegradable and has been proven to be a safe and non-toxic excipient in pharmaceutical formulations such as a dressing in wound healing, in tissue engineering, and for surface modification of implantable devices [2,7,8]. In addition, it can be easily manufactured into nanofiber, beads, micro- and nanoparticles, among other delivery systems [9].

Chitosan can be used as mucoadhesive polymer for drug delivery via various mucosal surfaces. The positive charge of chitosan molecule is considered to be the main factor responsible for its mucoadhesive properties; the electrostatic interactions between the mucus layer containing negatively charged mucin and positively charged chitosan are considered the reason for its good adhesion on the mucosal surfaces [10]. In addition to the electrostatic forces there are other possible contributing factors to its mucoadhesivness, such as its wettability, entanglement, possible interactions with the mucin from the weaker Van der Waal’s forces, and hydrogen bonding, as well as the hydrophobic interactions between the hydrophobic segments of the molecules. This enhanced bioadhesiveness will lead to increased retention time at the administration site, ensuring localized drug release and improved therapy. The use of chitosan in drug delivery systems has been extensive, both for systemic and localized drug delivery [4,11]; it has been shown to be a valuable excipient in tablets, emulsions, powders and gels providing a controlled release of the incorporated drug. On the smaller end of the scale chitosan has also been used in the development of chitosan-based nanoparticles, nanoemulsions and as a coating material for liposomes [12,13,14,15,16,17,18].

Local treatment with mucoadhesive drug delivery systems can offer several advantages, such as reduced administration frequencies, prolonged residence time and avoidance of disadvantages of systemic treatment. Additional advantage of chitosan as a mucoadhesive polymer is that it does not inactivate upon contact with mucin and its mucoadhesiveness does not weaken with time [19].

In respect to vaginal drug delivery systems, the main obstacles that need to be overcome for successful localized therapy are the great variations in the local pH and epithelial thickness depending on the age and hormone status, and a highly folded epithelial surface. Nanomedicine, particularly mucoadhesive nanopharmaceuticals, offers means of achieving a uniform distribution throughout the vaginal site [16,20]. Our group has been extensively studying the delivery systems able to improve local vaginal drug therapy. The mucoadhesiveness of chitosan is pH-dependent and stronger at the acidic pH providing an additional reason why we believe that chitosan has a great potential in vaginal delivery. We have recently developed several chitosan-based mucoadhesive drug delivery systems for local vaginal treatment [17,21,22]. The methods used to include/attach chitosan to the delivery systems varied from a simple one-pot preparation method, where chitosan was included in the first preparation step [21], to chitosan coating of the surface of preformed liposomes [17] or chitosan used as an excipient in pre-liposome tablets [22].

In respect to simplicity of the manufacturing conditions, the one-pot preparation method for production of chitosan-containing liposomes is particularly interesting [21]. The preparation process resulted in an* in situ* coating of the liposomes where it was hypothesized that the polymer is found both as a coating on the surface of the chitosan-containing liposomes and embedded in the aqueous compartment within the liposomes. In this study we wanted to further characterize this novel delivery system, particularly focusing on the mucoadhesiveness of the system and its ability to incorporate larger drug molecules, like biologicals. For that purpose we prepared chitosan-containing liposomes with two different model substances (fluorescein isothiocyanate dextran of Mw 4000 and 20,000 Da, FITC-dextran 4 and FITC-dextran 20, respectively). The ability of this type of liposomes to interact with mucin and, at the same time, provide sustained release of entrapped fluorescent substances was compared with the non-mucoadhesive (plain) and chitosan-coated liposomes containing the same dextran.

## 2. Results and Discussion

In order to achieve optimal treatment in local vaginal drug delivery it is important to provide a sufficient amount of the drug at the vaginal site for a sufficient amount of time [23]. Moreover, lower doses, drug targeting to the vaginal site, lower administration frequency may also lead to cost reduction of the therapy [24]. The important features of a drug delivery system directly contributing to the efficacy of the therapy are the drug load (entrapment of the drug within the carrier) and the mucoadhesion of the system, which will both ensure the increased concentration of the drug at the active site and its prolonged residence time [20]. In addition, the mucoadhesive delivery system needs to exhibit a predictable release of the entrapped/incorporated drug and be of a size that allows the system to reach the target tissue within the vaginal cavity [25]. These important characteristics were therefore the focus of this study.

### 2.1. Characterization of Liposomes

**Figure 1:**
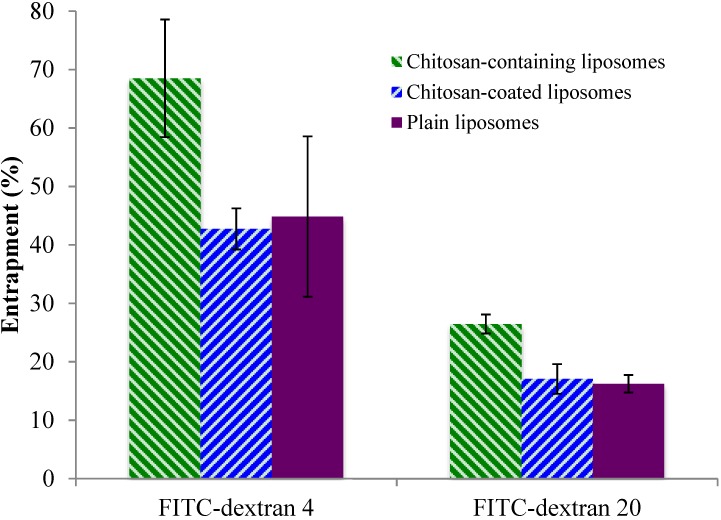
Entrapment of two FITC-dextrans in chitosan-containing liposomes, chitosan-coated liposomes, and plain liposomes. All values represent the mean ± SD (*n =* 3).

The entrapment of the model substances in three types of liposomes is presented in Figure 1. Both the low and high molecular weight FITC-dextrans were entrapped to the highest extent within chitosan-containing liposomes. The entrapment within the chitosan-coated and plain liposomes was similar for each type of FITC-dextran, and the pattern was consistent for both the low and high molecular weight FITC-dextrans. This may be explained by the fact that the liposomes that are the basis of the chitosan-coated liposomes are the same as the plain liposomes, except for the additional chitosan coating on their surface, and that the FITC-dextrans have been entrapped into the liposomes prior to the coating. The chitosan-containing liposomes are entirely different types of liposomes as they are formed in the presence of both chitosan and drug, in this case the model FITC-dextran. The presence of chitosan inside as well as outside the liposomes probably contributes to pulling more of the substance into the aqueous compartments of the liposomes. Both FITC-dextran and chitosan have a high number of hydrogen-bonding capable groups, which may contribute positively to pulling more FITC-dextrans into the liposomes; in addition, the chitosan embedded in the liposomal structure may disorganize the structure of the lipid bilayers and provide more room for the FITC-dextran inside the aqueous compartments of the chitosan-containing liposomes. As expected, the entrapment of FITC-dextran 20 was less than FITC-dextran 4 for all types of vesicles, which can be attributed to its larger molecular weight. However, both model substances were entrapped with rather high efficiencies indicating that the chitosan-containing liposomes can entrap sufficient amounts of larger molecules, such as biologicals, within their structure.

The liposomes prepared by the one-pot method are known to be larger than 1 micron with rather high polydispersity index (PI). They were also clearly of multilamellar nature [21]. To gain more control over the polydispersity of the samples, since this is expected to influence both the distribution of liposomes within vaginal cavity and the drug release rate, the sonication was applied. The sizes of the sonicated liposomes are best described by bimodal distributions where similarly sized liposomes are grouped in populations and the volume-weighted percentage of particles with a specific mean are calculated (Table 1). Chitosan-coated liposomes were the smallest of tested formulations, whereas the plain liposomes were the largest. Interestingly, liposomes containing FITC-dextran 20 were of smaller sizes than the same liposomes containing FITC-dextran 4. The smaller size of the liposomes containing FITC-dextran 20 can also be seen as a contributing factor to why the larger model substance was entrapped to a lower extent as compared to the FITC-dextran 4; smaller liposomes have less available aqueous part for accommodation of hydrophilic molecules. Rather unexpected results were the size distributions of plain liposomes. However, similar findings that polymer-coated liposomes were smaller than non-coated liposomes were reported earlier [26,27]. The reason behind this observation could be that chitosan is known to form a cage-like steric barrier that protects liposomes from aggregation, whereas in the case of non-coated liposomes the agglomeration can occur [26].

**Table 1:** Size distributions of liposomes. All values represent the mean size ± SD, and are volume-weighted (%) bimodal distribution (*n =* 3).

Type of Liposomes	Peak 1 *		Peak 2 *		PI
	Size (nm)	%	Size (nm)	%	
*FITC-dextran 4*					
Chitosan-containing	76 ± 40	20 ± 7	287 ± 48	79 ± 9	0.30 ± 0.01
Chitosan-coated	48 ± 25	69 ± 3	197 ± 27	21 ± 3	0.35 ± 0.15
Plain	56 ± 20	16 ± 13	337 ± 53	85 ± 13	0.36 ± 0.08
*FITC-dextran 20*					
Chitosan-containing	50 ± 19	29 ± 7	257 ± 42	64 ± 8	0.33 ± 0.01
Chitosan-coated	27 ± 4	26 ± 9	99 ± 18	74 ± 9	0.34 ± 0.01
Plain	51 ± 3	39 ± 2	219 ± 3	54 ± 24	0.37 ± 0.05

* The values are shown as a Nicomp distribution, which gave the best fit for the measured data (Fit error <1.5; residual error <10).

The zeta potential of the plain liposomes, regardless of the type of the entrapped FITC-dextran, was close to neutral (0.93 mV), which is expected since the lipid used to form vesicles is neutral. The chitosan containing formulations, chitosan-containing and chitosan-coated liposomes, exhibited a positive zeta potentials (2.45 and 6.73 mV, respectively) reflecting the positive charge of the surface-available chitosan.

### 2.2. Surface-Available Chitosan

**Figure 2:**
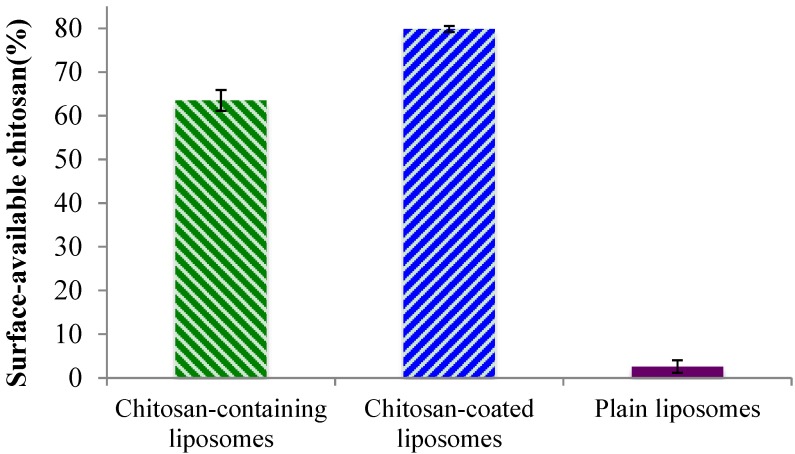
Percentage of surface-available chitosan determined in chitosan-containing, chitosan-coated liposomes, and plain liposomes. All values represent the mean ± SD (*n =* 3).

Although the zeta potentials indicated the presence of chitosan on the liposomal surface for both the chitosan-containing and chitosan-coated liposomes, we wanted to confirm that chitosan is indeed available to interact with mucin and thus ensure the system’s bioadhesiveness. In the case of the chitosan-containing liposomes, where chitosan was present during the formation of the liposomes, it is expected that a proportion of the polymer is lodged inside the lamellar structure of the liposomes. Therefore, to prove this hypothesis, the availability of chitosan on the surfaces of all liposomes was evaluated. As can be seen in Figure 2, the plain liposomes did not exhibit any (or in a negligent amount) surface-available chitosan. The small percentage detected can be due to a limitation of the test. The chitosan-containing and chitosan-coated liposomes exhibited a high degree of surface-available chitosan; the chitosan-coated vesicles contained significantly (*p* < 0.01) more chitosan on their surface (80%) as compared to the chitosan-containing liposomes (approx. 65%). Considering that for the chitosan-coated liposomes all, or most of the chitosan, should be surface-available, this finding was as expected. The fact that about 35% of chitosan was not surface-available in the chitosan-containing liposomes indicated that parts of chitosan are indeed embedded within this type of liposomes, and proved our initial hypothesis. These findings are also in agreement with the zeta potentials measured on liposomal surfaces where the chitosan-coated liposomes exhibited higher zeta potential.

### 2.3. Mucin-Binding Properties of Liposomes

**Figure 3:**
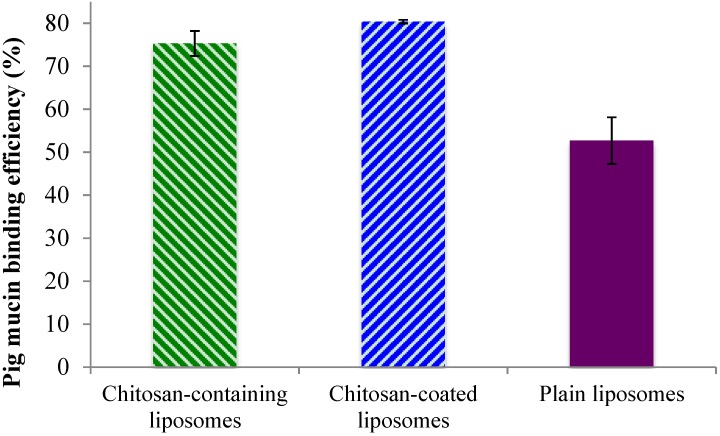
Binding efficacy of the liposomes to porcine mucin. All values represent the mean ± SD (*n =* 3).

After confirming that there are high amounts of surface-oriented chitosan available for possible interaction with mucin, both on the surface of the chitosan-containing and chitosan-coated liposomes, an* in vitro* mucin test was applied to confirm the system’s adhesiveness. The binding efficiency of the liposomes to the model porcine mucin (PM) was used to demonstrate the mucin-binding capability of the formulations and to estimate the mucoadhesive behavior. The chitosan-coated liposomes exhibited the highest PM binding efficiency, closely followed by the chitosan-containing liposomes, while the plain liposomes, as expected, exhibiting lower mucoadhesiveness (Figure 3). These findings were in direct agreement with the chitosan surface availability data (Figure 2) where the chitosan-coated liposomes were shown to have more available chitosan on the surface. However, even though the chitosan-containing and chitosan-coated liposomal formulations were significantly different (*p* < 0.05) regarding the mucin-binding capacity, this was less pronounced than the difference in the amount of surface available chitosan. The plain liposomes exhibited the PM binding efficiency of about 50%, which is significantly less (*p* < 0.001) than the other two liposomal formulations. One can argue that plain liposomes should have negligible mucin-binding capacity; however due to the ultracentrifugation applied to separate liposomes from bound mucin, it is possible that some plain liposomes interacted physically with the mucin without the actual electrostatic interactions that were targeted in the test. It is also possible that there is a hydrophobic interaction between the liposomes and mucin that leads to the findings in Figure 3. Our findings are also comparable to the results reported earlier for the sonicated plain liposomes [27].

### 2.4. In Vitro Release of FITC-Dextrans from Liposomes

**Figure 4:**
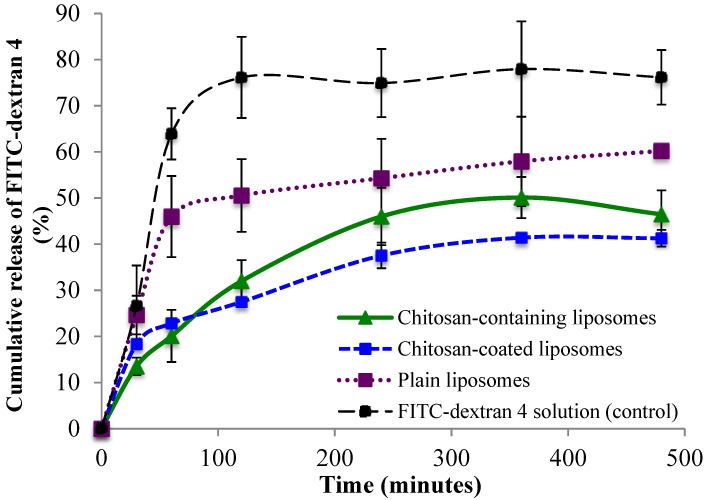
Cumulative release of FITC-dextran 4 from chitosan-containing liposomes, chitosan-coated liposomes, and plain liposomes. All values represent the mean ± SD (*n =* 3).

**Figure 5:**
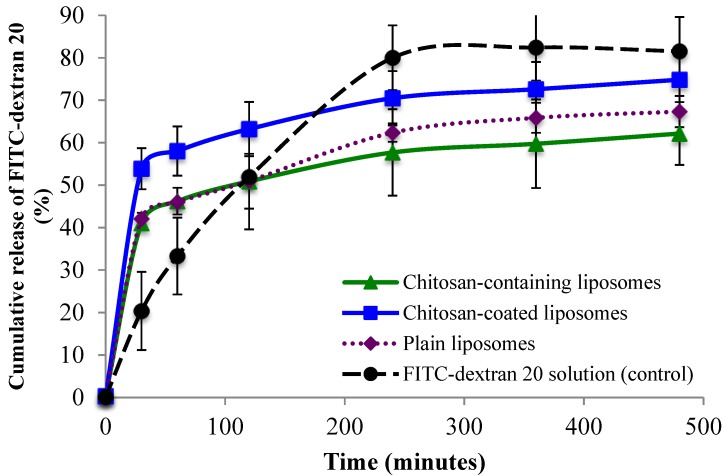
Cumulative release of FITC-dextran 20 from chitosan-containing liposomes, chitosan-coated liposomes, and plain liposomes. All values represent the mean ± SD (*n =* 3).

Cumulative release of FITC-dextran 4 and FITC-dextran 20 from all three liposomal formulations is shown in Figure 4 and Figure 5, respectively. All three types of delivery systems released FITC-dextran 4 in a sustained manner as compared to the FITC-dextran 4 solution (Figure 4). The chitosan-containing liposomes were found to sustain the initial release to a greater extent than the chitosan-coated liposomes, however after 2 h the release of FITC-dextran 4 was slower from the chitosan-coated compared to chitosan-containing liposomes although not on significant level. The release of the high molecular weight FITC-dextran 20 (Figure 5) was found to be faster than the release of low molecular FITC-dextran 4. Again the control (FITC-dextran 20 solution) exhibited the highest cumulative release; however in this case the chitosan-containing liposomes sustained the release of FITC-dextran 20 to the greatest extent among the tested formulations. Interestingly, the chitosan-coated liposomes released more FITC-dextran 20 than the plain liposomes (Figure 5), which is exactly the opposite behavior as found for the low molecular weight FITC-dextran 4. Another interesting observation was the fast initial release of FITC-dextran from all liposomal formulations. It seems that the chitosan-coated liposomes provided an initial burst release of the high molecular weight fluorescent marker. One possible explanation can be that the rather large molecule of FITC-dextran 20 (20,000 Da) was not only entrapped but also embedded between the vesicle bilayers close to the outer bilayer and was released by a rapid diffusion at the start of the release study. In addition, the smaller liposomal size, and thus the larger total surface area of liposomes containing FITC-dextran 20 could facilitate its faster release as compared to the release of FITC-dextran 4.

Based on the surface availability of chitosan on the chitosan-containing liposomes, and the ability to bind to mucin, this type of liposomes offers the potential to adhere to the vaginal mucosa and reside at the vaginal site for a prolonged period of time to ensure sufficiently high amounts of the drug at the site of action. The entrapment of large molecular weight model substances in the chitosan-containing liposomes was superior to entrapment of the same substances in the chitosan-coated liposomes. Moreover, the release of incorporated model substances of higher molecular weights (4000 and 20,000 Da) indicates that the chitosan-containing liposomes will release the incorporated material (e.g., drug) in a sustained manner. The fate of intravaginally administered drugs can be seen as a multi-compartment interactive event where the effects of the delivery system, the amount and viscosity of vaginal fluid, the presence of semen, the epithelium conditions and disease state need to be considered when optimizing the formulation [28]. Additional advantage of chitosan is its non-toxicity, as it is expected that chitosan-based delivery systems will not cause vaginal irritation. Very recently, the indications that chitosan can disrupt the bacterial biofilms in bacterial vaginosis have been reported, which would strengthen the potentials of chitosan as vaginal mucoadhesive delivery system [29].

## 3. Experimental Section

### 3.1. Materials

Soy phosphatidylcholine (Lipoid S100, Lipoid GmbH, Ludwigshafen, Germany) was a generous gift by Lipoid GmbH. Chitosan (60,000 Da; 77% degree of deacetylation (DD)), two types of fluorescein isothiocyanate dextran (FITC-dextran 4 and FITC-dextran 20 corresponding Mw 4000 and 20,000, respectively), mucin from porcine stomach type II, Triton X, methanol and n-propanol were all products of Sigma Aldrich Inc. (Steinheim, Germany). Cibacron Brilliant Red 3B-A was purchased from Santa Cruz Biotechnology Inc. (Santa Cruz, CA, USA). Sepharose CL-4B gel was ordered from Pharmacia Bioteck AB (Uppsala, Sweden). All other chemicals used in the experiments were of analytical grade.

### 3.2. Preparation of Vesicles

#### 3.2.1. Preparation of Chitosan-Containing Liposomes

Chitosan-containing liposomes were prepared by the one-pot preparation method previously developed in our research group [21]. Briefly, Lipoid S100 (SPC, 200 mg) was dissolved in an adequate amount of methanol. The solvent was evaporated using a rotoevaporator system (Büchi rotavapor R-124, with vacuum controller B-721, Büchi vac V-500, Büchi Labortechnik, Flawil, Switzerland) under a vacuum at 45 °C. The resulting film was redispersed with 100 μL of n-propanol by help of a micro syringe pipette (Hamilton, Bonaduz, Switzerland). The dispersion was needle-injected into 2 mL of aqueous media containing 0.17% (w/w) chitosan in 0.1% (v/v) acetic acid and either FITC-dextran 4 or FITC-dextran 20 (42.0 mg) and stirred for 2 h at room temperature (23 °C). The dispersion was left in a refrigerator overnight prior to vesicle size reduction and characterization.

#### 3.2.2. Plain Liposomes

Plain, non-mucoadhesive liposomes, were prepared under the same conditions using the same lipid composition to prepare the film, which was subsequently redispersed and injected into aqueous solution of either FITC-dextran 4 or FITC-dextran 20. The dispersion was left in a refrigerator overnight prior to vesicle size reduction and characterization.

#### 3.2.3. Vesicle Size Reduction

The chitosan-containing and plain (non-mucoadhesive) liposomes were reduced to a smaller size by sonication using a Sonics High Ultrasonic Processor (Sigma-Aldrich Chemie GmbH, Steinheim, Germany). Prior to sonication, the samples were diluted to a suitable volume (5 mL) with distilled water and sonicated for 45 s using an ice bath to prevent heating of the samples.

#### 3.2.4. Chitosan-Coated Liposomes

Coating of sonicated plain FITC-dextran containing liposomes was performed by a previously reported method [17]. In brief, the chitosan solution (0.1% w/v) in glacial acetic acid (0.1% v/v) was added drop-wise to an equal volume of liposomes under the controlled magnetic stirring at room temperature for 1 h. Upon completion of stirring, the dispersion was left in a refrigerator overnight.

### 3.3. Entrapment Efficiency

In order to remove the unentrapped FITC-dextrans from liposomes two different separation methods were used, depending of the molecular weight of the model substance. For the liposomes containing FITC-dextran 4, dialysis in a dialysis membrane (Mw cut off: 12–14,000 Daltons; Medicell International Ltd., London, UK) against distilled water was applied for 24 h at room temperature. For liposomes containing FITC-dextran 20, a column separation on a Sepharose CL-4B gel was used.

The entrapment efficiency of the liposomal formulations was determined by fluorescence measurements using a Polarstar flourimeter (Fluostar, BMG Technologies, Offenburg, Germany) on excitation and emission wavelengths of 485 and 520 nm, respectively. To dissolve lipid, liposomal samples were pretreated by addition of 10% (v/v) of Triton X in a volume ratio of 1:1. Standard curves for both FITC-dextrans in water and FITC-dextrans in aqueous Triton X solutions were prepared and used for the fluorescence determination.

### 3.4. Particle Size Analysis

The size distributions were measured by photon correlation spectroscopy using a Submicron Particle-sizer (Model 360, Nicomp, Santa Barbara, CA, USA). To avoid possible interference caused by dust particles, test tubes were pre-rinsed with distilled water and bath-sonicated for 10 min. In addition, all sample preparations were performed in a laminar airflow bench. The liposomal samples were diluted with filtered (0.2 μm Milipore filters) distilled water to provide appropriate count intensity (approx. 250–350 kHz) and measured in three parallels (run time 10 min at 23 °C). Both Gausssian and Nicomp algorithms were fitted to the experimental data to find the distribution that best describes the particle population [30]. As the fit error was found to be smaller than 1.5, and the residual error was smaller than 10, Nicomp distribution was selected. The volume-weighted distribution was used to determine the mean diameter and PI of all samples.

### 3.5. Zeta Potential Determination

The zeta potential of all liposomes was measured on a Malvern Zetasizer Nano ZS (Malvern Instruments Ltd, Oxford, UK). The instrument was calibrated throughout the measurements using the Malvern zeta potential transfer standard (−50 ± 5 mV). The samples were diluted in filtered water until an appropriate count rate was achieved and measured in a measuring cell. All measurements were performed at 23 °C and the results represent an average of at least three independent measurements [16].

### 3.6. Determination of Surface-Available Chitosan

To determine the surface-available chitosan the colorimetric method originally reported by Muzzarelli [31] was applied. Glycine buffer (pH 3.2) was prepared by dissolving 1.87 g of glycine and 1.46 g of NaCl in 250 mL of distilled water; an aliquot of 81 mL was further diluted with 0.1 M HCl to a final volume of 100 mL. Cibacron Brilliant Red 3B-A (150 mg) was dissolved in 100 mL of distilled water. The dye solution (5 mL) was further diluted to 100 mL with the glycine buffer. Liposomal suspensions were diluted with distilled water to desirable concentration (1:2, v/v) before 3 mL of the final dye solution was added. UV absorbance was measured photometrically at 575 nm (Agilent Technologies, Santa Clara, CA, USA). The surface-available chitosan was calculated using the following equation:

$$Surface-available\;chitosan=\frac{C_{s}}{C_{c}}\times 100$$

where Cs is the concentration of surface-available chitosan in the sample and Cc is the concentration of chitosan used to prepare the liposomal formulations.

A standard curve was made by suspending chitosan powder (0.5 g) in 50 mL of distilled water. After 30 min at room temperature, 2.0 mL of glacial acetic acid (99.8% w/w) was added. An additional 50 mL of distilled water was added to acidic chitosan solution, before the final dilution with distilled water provided a final concentration of 0.5 g/L. Standard solutions were made by diluting the chitosan solution with glycine buffer to desired concentrations.

### 3.7. Mucin-Binding Test as Indicator of Mucoadhesiveness

The mucoadhesive properties were determined by the method developed by Pawar* et al.* [32] and modified in our group [27]. The porcine mucin (PM; 400 μg/mL) was hydrated in the phosphate buffer (0.05 M, pH 7.4), the suspension mixed with the vesicle suspension (1:1, v/v) and the mixture incubated at room temperature (23 °C) for 2 h prior to centrifugation for 60 min at 216,000× *g* and 10 °C (Optima LE-80; Beckman Instruments, Palo Alto, CA, USA). Absorbance of the remaining free PM in the supernatants was measured by UV spectrophotometry (Microtitre plate reader; Spectra Max 190 Microplate, Spectrophotometer Molecular Devices, Sunnyvale, CA, USA) at 251 nm. The mucoadhesiveness was expressed as PM binding efficiency calculated by the following equation:

$$PM\;binding\;eff.\;=\;\left(\frac{C_{0}-C_{S}}{C_{0}}\right)\times 100$$

where C_0_ is the initial concentration of PM used for incubation (400 μg/mL) and C_S_ is the measured concentration of free PM in the supernatant after removal of the liposome-bound PM. The standard curve was determined from the standard PM solutions in the phosphate buffer made by diluting the PM stock solution to 40, 80, 120, 160, 200, 240, 280, and 320 μg/mL, respectively.

### 3.8. In Vitro Release Studies

Release studies were performed using the Franz diffusion cells (PermeGear, Hellertown, PA, USA) with the heating circulator (Julabo Labortechnik F12-ED, Seelback, Germany) maintaining the temperature at 37 °C. The cells with 12 mL volume acceptor chambers and a diffusion area of 1.77 cm^2^ were used in* in vitro* studies based on the method by Hurler* et al.* [33] Polyamide membranes (0.2 μm pore size, Sartorius polyamide membrane; Sartorius AG, Gröttingen, Germany) were used. The formulations were added to the donor compartment in a volume of 600 μL. The acceptor chambers were filled with distilled water, and kept at 37 °C. Samples (500 μL) from the acceptor medium were taken at 30, 60, 120, 240, 360 and 480 min, and replaced with the fresh medium. Both the sampling port and the donor chamber were covered with quadruple layers of parafilm to prevent evaporation. Quantification of released fluorescent markers was determined based on the flourimetric measurements at excitation and emission wavelengths of 485 and 520 nm, respectively. All experiments were carried out in triplicate.

### 3.9. Statistical Evaluation

The student’s *t*-test was used for comparison of two means. A significance level of *p* < 0.05 was considered to be appropriate.

## 4. Conclusions

The mucoadhesive nanosize delivery systems, the chitosan-containing liposomes, were shown to entrap/incorporate higher amounts of the fluorescent model substances of different molecular weight (4000 and 20,000 Da) as compared to conventional plain and chitosan-coated liposomes. The higher entrapment can be explained by the embedding of chitosan also within the lamellar structure of the liposomes and not only on the surface as proven in the surface-availability tests. The chitosan-containing liposomes were also able to ensure sustained release of entrapped material. The ability of surface-available chitosan to interact with mucus was confirmed indicating system’s potential to prolong the residence time at the vaginal site.

## References

[1] Prashant K.V.H., Tharanathan R.N. (2007). Chitin/chitosan: Modifications and their unlimited application potential- an overview. Trends Food Sci. Technol..

[2] Pal K., Behera B., Roy S., Ray S.S., Thakur G. (2013). Chitosan based delivery systems on a length scale: Nano to macro. Soft Mater..

[3] Bernkop-Schnuerch A., Duennhaupt S. (2012). Chitosan-based drug delivery systems. Eur. J. Pharm. Biopharm..

[4] Uchgebu I.F., Carlos M., McKay C., Hou X., Schaetzlein A.G. (2014). Chitosan amphiphiles provide new drug delivery opportunities. Polym. Int..

[5] Dash M., Chiellini F., Ottenbrite R.M., Chiellini E. (2011). Chitosan-A versatile semisynthetic polymer in biomedical applications. Prog. Polym. Sci..

[6] Kean T., Thanou M. (2010). Biodegradation, biodistribution and toxicity of chitosan. Adv. Drug Deliv. Rev..

[7] Kim I.-Y., Seo S.-J., Moon H-S., Yoo M.-K., Park I.-Y., Kim B.-C., Cho C.-S. (2008). Chitosan and its derivatives for tissue engineering applications. Biotechnol. Adv..

[8] Bhattarai N., Gunn J., Zhang M. (2010). Chitosan-based hydrogels for controlled, localized drug delivery. Adv. Drug Deliv. Rev..

[9] Jayakumar R., Menon D., Manzoor K., Nair S.V., Tamura H. (2010). Biomedical applications of chitin and chitosan based nanomaterials—A short review. Carbohyd. Polym..

[10] Singla A.K., Chawla M. (2001). Chitosan: Some pharmaceutical and biological aspects—An update. J. Pharm. Pharmacol..

[11] Perioli L., Ambrogi V., Venezia L., Pagano C., Ricci M., Rossi C. (2008). Chitosan and modified chitosan as agents to improve performances of mucoadhesive vaginal gels. Colloids Surf. B Biointerfaces.

[12] Li N., Zhuang C., Wang M., Sun X., Nie S., Pan W. (2009). Liposomes coated with low molecular weight chitosan and its potential use in ocular drug delivery. Int. J. Pharm..

[13] Zaru M., Manca M.-L., Fadda A.M., Antimisiaris S.G. (2009). Chitosan-coated liposomes for delivery to lungs by nebulization. Colloids Surf. B Biointerfaces.

[14] Calderon L., Harris R., Cordoba-Diaz M., Elorza M., Elorza B., Lenoir J., Andriaens E., Remon J.P., Heras A., Cordoba-Diaz D. (2013). Nano and microparticulate chitosan-based systems for antiviral topical delivery. Eur. J. Pharm. Sci..

[15] Casettari L., Illum L. (2014). Chitosan in nasal delivery systems for therapeutic drugs. J. Control. Release.

[16] Vanić Ž., Škalko-Basnet N. (2014). Mucosal nanosystems for improved topical drug delivery: Vaginal route of administration. J. Drug. Del. Sci. Technol..

[17] Jøraholmen M.W., Vanić Ž., Tho I., Škalko-Basnet N. (2014). Chitosan-coated liposomes for topical vaginal therapy: Assuring localized drug effect. Int. J. Pharm..

[18] Berginc K., Suljaković S., Škalko-Basnet N., Kristl A. (2014). Mucoadhesive liposomes as new formulations for vaginal delivery of curcumin. Eur. J. Pharm. Biopharm..

[19] Valenta C. (2005). The use of mucoadhesive polymers in vaginal delivery. Adv. Drug Delivery Rev..

[20] Vanić Ž., Škalko-Basnet N. (2013). Nanopharmaceuticals for improved topical vaginal therapy: Can they deliver?. Eur. J. Pharm. Sci..

[21] Andersen T., Vanić Ž., Flaten G.E., Mattsson S., Tho I., Škalko-Basnet N. (2013). Pectosomes and chitosomes as delivery systems for metronidazole: The one-pot preparation method. Pharmaceutics.

[22] Vanić Ž., Planinšek O., Škalko-Basnet N., Tho I. (2014). Tablets of pre-liposomes govern* in situ* formation of liposomes: Concept and potential of the novel drug delivery system. Eur. J. Pharm. Biopharm..

[23] Hainer B.L., Gibson M.V. (2011). Vaginitis: Diagnosis and treatment. Am. Fam. Physician.

[24] Andrews G.P., Laverty T.P., Jones D.S. (2009). Mucoadhesive polymeric platforms for controlled drug delivery. Eur. J. Pharm. Biopharm..

[25] Das Neves J., Bahia M.F., Amiji M.M., Sarmento B. (2011). Mucoadhesive nanomedicines: Characterization and modulation of mucoadhesion at the nanoscale. Expert Opin. Drug Deliv..

[26] Tan H.W., Mishran M. (2012). Characterization of fatty acid liposome coated with low molecular-weight chitosan. J. Liposome Res..

[27] Naderkhani E., Erber A., Škalko-Basnet N., Flaten G.E. (2014). Improved permeability of acyclovir: Optimization of mucoadhesive liposomes using the Phospholipid Vesicle-Based Permeation Assay. J. Pharm. Sci..

[28] Katz D.F., Gao Y., Kang M. (2011). Using modeling to help understand vaginal microbicide functionality and create better products. Drug Deliv. Transl. Res..

[29] Kandimalla K.K., Borden E., Omtri R.S., Boyapati S.P., Smith M., Lebby K., Mulpuru M., Gadde M. (2013). Ability of chitosan gels to disrupt bacterial biofilms and their applications in the treatment of bacterial vaginosis. J. Pharm. Sci..

[30] Di Cagno M., Styskala J., Hlavac J., Brandl M., Bauer-Brandl A., Škalko-Basnet N. (2011). Liposomal solubilization of new 3-hydroxy-quinolinone derivatives with promising anticancer activity: A screening method to identify maximum incorporation capacity. J. Liposome Res..

[31] Muzzarelli R.A.A. (1998). Colorimetric determination of chitosan. Anal. Biochem..

[32] Pawar H., Douroumis D., Boateng J.S. (2012). Preparation and optimization of PMAA-chitosan-PEG nanoparticles for oral drug delivery. Colloids Surf. B Biointerfaces.

[33] Hurler J., Berg O.A., Skar M., Conradi A.H., Johnsen P.J., Škalko-Basnet N. (2012). Improved burns therapy: Liposomes-in-hydrogel delivery system for mupirocin. J. Pharm. Sci..

